# Broad Neutralization Capacity of an Engineered Thermostable Three-Helix Angiotensin-Converting Enzyme 2 Polypeptide Targeting the Receptor-Binding Domain of SARS-CoV-2

**DOI:** 10.3390/ijms252212319

**Published:** 2024-11-16

**Authors:** Davide Cavazzini, Elisabetta Levati, Saveria Germani, Bao Loc Ta, Lara Monica, Angelo Bolchi, Gaetano Donofrio, Valentina Garrapa, Simone Ottonello, Barbara Montanini

**Affiliations:** 1 Laboratory of Biochemistry and Molecular Biology, Department of Chemistry, Life Sciences and Environmental Sustainability, University of Parma, 43124 Parma, Italy; davide.cavazzini@unipr.it (D.C.); elisabetta.levati@unipr.it (E.L.); angelo.bolchi@unipr.it (A.B.); simone.ottonello@unipr.it (S.O.); 2 Preclinics GMBH, 14482 Potsdam, Germany; sg@preclinics.com (S.G.); blt@preclinics.com (B.L.T.); lm@preclinics.com (L.M.); 3 Interdepartmental Research Centre Biopharmanet-Tec, University of Parma, 43124 Parma, Italy; 4 Department of Medical Veterinary Science, University of Parma, 43126 Parma, Italy; gaetano.donofrio@unipr.it; 5 Preclinics Italia srl, 43121 Parma, Italy; vg@preclinics.com

**Keywords:** SARS-CoV-2, COVID-19, blocking peptides, spike-ACE2 interface, RBD, antiviral blockers, ACE2 decoy, spike-ACE2 interaction inhibitor

## Abstract

The mutational drift of SARS-CoV-2 and the appearance of multiple variants, including the latest Omicron variant and its sub-lineages, has significantly reduced (and in some cases abolished) the protective efficacy of Wuhan spike-antigen-based vaccines and therapeutic antibodies. One of the most functionally constrained and thus largely invariable regions of the spike protein is the one involved in the interaction with the ACE2 receptor mediating the cellular entry of SARS-CoV-2. Engineered ACE2, both as a full-length protein or as an engineered polypeptide fragment, has been shown to be capable of preventing the host-cell binding of all viral variants and to be endowed with potent SARS-CoV-2 neutralization activity both in vitro and in vivo. Here, we report on the biochemical and antiviral properties of rationally designed ACE2 N-terminal, three-helix fragments that retain a native-like conformation. One of these fragments, designated as PRP8_3H and produced in recombinant form, bears structure-stabilizing and binding-affinity enhancing mutations in α-helix-I and in both α-helix I and II, respectively. While the native-like, unmodified three α-helices ACE2 fragment proved to be thermally unstable and without any detectable pseudovirion neutralization capacity, PRP8_3H was found to be highly thermostable and capable of binding to the SARS-CoV-2 spike receptor-binding domain with nanomolar affinity and to neutralize both Wuhan and Omicron spike-expressing pseudovirions at (sub)micromolar concentrations. PRP8_3H thus lends itself as a highly promising ACE2 decoy prototype suitable for a variety of formulations and prophylactic applications.

## 1. Introduction

The SARS-CoV-2 pandemic has prompted the search and development of different strategies aimed at limiting virus spreading [1,2]. While vaccines [3,4] and neutralizing monoclonal antibodies [5] were initially effective as COVID-19 treatments, each of the successive waves of so-called variants of concern (VOC), especially the latest Omicron variant, displayed a steadily increasing evasion capacity against vaccine-induced and therapeutic antibodies [6,7,8] and progressively limited the effectiveness of these immunological approaches [6,9,10,11]. This emphasized the difficulty of continuously updating vaccines and antibodies and the need for conceptually diverse therapeutic strategies that might be effective against current and future viral variants. 

SARS-CoV-2 infection starts with the binding of the spike receptor binding domain (RBD) to the membrane-bound angiotensin-converting enzyme 2 (ACE2) [12,13,14], the host receptor used by the virus for cellular internalization. While additional co-receptors have recently been identified [15,16], ACE2, which is involved in blood pressure homeostasis and binds the spike RBD with nanomolar affinity [17], is still recognized as the main host receptor for SARS-CoV-2 cellular entry [12,13,18,19]. Its three-dimensional structure has been determined at high resolution, both alone and in complex with different RBD spike variants [14,17,19,20,21]. Researchers have been targeting the ACE2-spike interaction to prevent SARS-CoV-2 infection. Most neutralizing antibodies present in SARS-CoV-2 immune sera bind to the spike RBD [22,23], whose receptor binding motif (RBM) harbors the majority of viral mutations responsible for humoral immunity evasion [24]. This prompted the development of the alternative ACE2 decoy approach as a means to sequester the virus and prevent its cellular internalization by interaction with an invariant region of the RBD [25]. In fact, while viral evolution can fix RBD mutations that confer resistance to otherwise neutralizing antibodies, mutational drift is constrained by the requirement to preserve tight binding to ACE2 as an essential prerequisite for viral infection capacity [26,27]. Natural and modified ACE2-derived polypeptides [28,29,30,31], corresponding to the entire receptor ectodomain [32,33] in a monomeric (Ig-Fc-fused) or artificially multimerized form as well as to specific ACE2 fragments [31], have been shown to act as virus sequestering decoys and to inhibit infection by several SARS-CoV-2 lineages [33,34,35,36,37]. Various amino acid substitutions, mainly located within the N-terminal helices of ACE2, significantly improved RBD binding affinity and led to in vitro neutralizing activities against multiple SARS-CoV-2 lineages, including the most recent Omicron VOCs, up to 300-fold higher than that of the wild-type receptor [25,38,39,40,41,42]. Most of the intermolecular contacts with the RBM portion of the spike RBD are located on the N-terminal α-helices I and II and on a short β-sheet of ACE2 [20]. Based on this observation, a rational minimization approach has led to the identification of various virus-neutralizing ACE2 fragments. These can include up to three ACE2 N-terminal α-helices (I to III) [43,44], but they all comprise α-helix I, which harbors most of the RBD interacting residues. However, a polypeptide only comprised of the native (unmodified) ACE2 α-helix I displayed low RBD affinity and no antiviral activity, due to intrinsic structural instability and disorder [45,46]. Although computational analysis predicted a native conformation for a two-helix ‘mini-protein’ thanks to stabilizing interactions between α-helices I–II [44,47], the wild-type three-helix N-terminal fragment of ACE2 displayed a limited thermal stability at 37 °C [48]. Peptide stapling as well as targeted modifications resulted in mono-helical (α-helix I) decoys with enhanced stability [49,50]. In particular, the introduction of a few hydrophobic amino acid substitutions strengthened helical folding in solution and conferred an enhanced in vitro neutralization capacity to the synthetic α-helix I “P8” peptide fragment [50]. Other spike decoys, sharing limited or no structural similarity to ACE2 yet neutralization-competent and capable of binding to specific RBM regions, have been artificially engineered. These include, for example, de novo designed mini-proteins such as CTC-445 [51] and LCB1 [52,53] that mimic a specific portion of the ACE2 interface and bind the spike RBD with high-affinity. These completely artificial decoys, which were endowed with a high neutralization capacity in vitro, also displayed a fairly strong prophylactic and therapeutic efficacy in animal models of SARS-CoV-2 infection [51,53,54]. However, RBD mutations at the ACE2-spike interface were found to significantly curtail the neutralizing capacity of the LCB1 mini-protein against Omicron variants [53]. Moreover, decoys based on the natural ACE2 protein or its engineered derivatives are expected to be considerably less immunogenic than their de novo designed artificial counterparts. 

While existing approaches and therapeutic strategies primarily utilize entire ACE2-based ectodomain for SARS-CoV-2 neutralization, recent advances in protein engineering have expanded the repertoire of ACE2-derived molecules [28,29,30,31]. Yet, the potential of multi-helical structures remains largely unexplored. Based on the above considerations, we focused on an engineered three-helix mini-protein derived from the N-terminal region (α-helices I–III) of ACE2. Our novel three-helix rational design addresses the key limitations of previous approaches by optimizing binding interface presentation, while enhancing structural stability through inter-helical interactions. This architecture represents a significant departure from conventional ACE2-based inhibitors and may provide a new framework for therapeutic protein design. We examined the biochemical and anti-viral properties of our polypeptide, with special attention paid to the effect of mutations predicted to stabilize the polypeptide as well as additional mutations expected to increase its binding affinity to the spike RBD. Our three-helix decoy was found to neutralize SARS-CoV-2 Wuhan and Omicron pseudovirions. Its marked thermostability and ease of production in recombinant form make it amenable to different prophylactic applications, pharmaceutical formulations, and modes of delivery. 

## 2. Results

### 2.1. Design of an Engineered Tri-Helical ACE2 Polypeptide Fragment

Our aim was to construct an ACE2 decoy fragment with a native-like conformation and high thermal stability, yet bearing minimal residue modifications compared to the corresponding portion of the wild-type ACE2 protein. Such a fragment should closely mimic the structure and main intermolecular interactions of the ACE2-RBD interface in order to prevent viral escape mutations and unwanted anti-decoy immune responses.

A rational re-design of excised α-helices is often needed to retain native secondary and tertiary conformations and thus the ability to bind the desired target [55]. Due to the contacts among helices α-I, α-II, and α-III [47], the presence of two or more ACE2 N-terminal α-helices instead of one is expected to improve overall mini-protein stability and bent shape. In fact, a bi-helical ACE2 (α-I, α-II) mini-protein was predicted by computational analysis to be more stable in an aqueous environment than the mono-helical (α-I only) form [44]. However, given the extended length of α-helices I and II, and the limited stability of the overall three-coil fragment [48], we decided to substitute eight amino acids of helix α-I, not involved in the ACE2-RBD interaction, with hydrophobic amino acid residues previously shown to significantly improve the conformational stability of the one-helix P8 peptide [50]. The predicted structure of the ACE2 αI–III helices bearing the P8 mutations does not indicate any particular steric hindrance at the α-helix II interface compared to the wild-type ACE2 structure (Figure S1A,B). Instead, additional stabilizing contacts are predicted to form within α-helix III as a consequence of the α-I P8 mutations.

**Figure 1:**
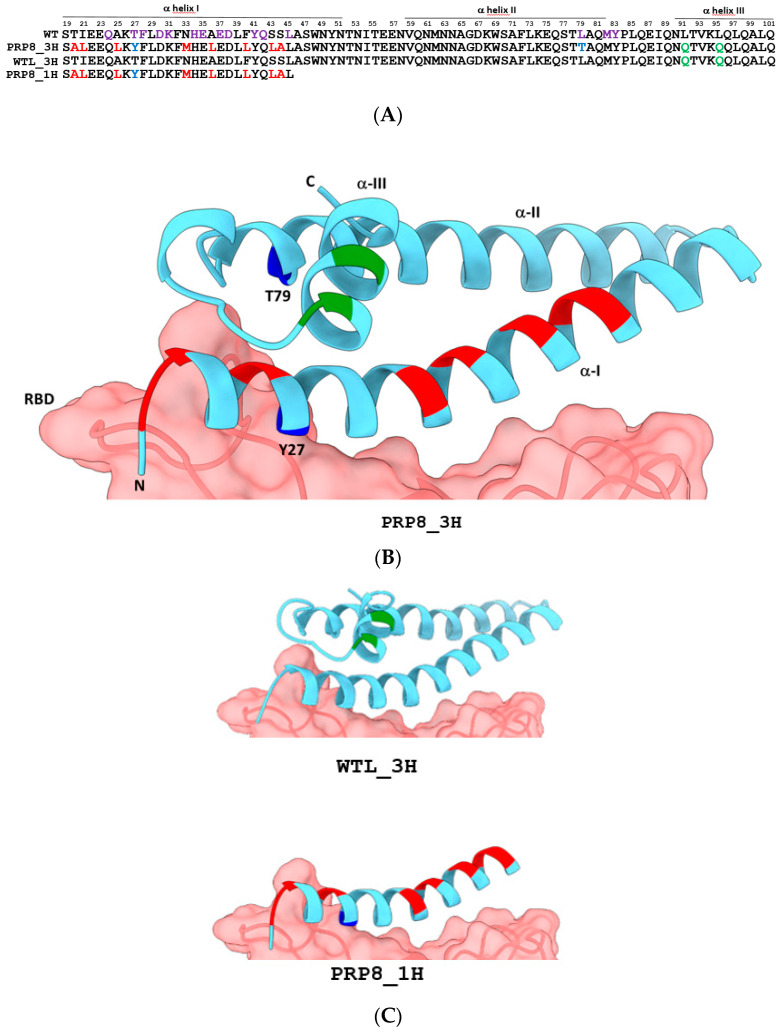
Amino acid sequences and predicted structures of the three ACE2 polypeptide fragments investigated in this study. (**A**) Amino acid sequences of the engineered ACE2 polypeptides aligned with the corresponding sequence of the human ACE2 receptor (WT). In WT ACE2 (upper sequence), the amino acid residues involved in SARS-CoV-2 RBD binding are shown in violet. In the engineered ACE2 polypeptide fragment sequences (indicated as PRP8_3H, WTL_3H, and PRP8_1H, for the three-helical engineered and wild-type-like, and the mono-helical P8 polypeptides, respectively; see text for details), invariant, wild-type identical amino acids are in black, while mutated residues are shown in red, blue, or green in the case of P8, V2.4 affinity-enhancing, and helix-III mutated residues, respectively. (**B**) Three-dimensional structure of the PRP8_3H decoy predicted by the SWISS-MODEL server, represented as a light blue ribbon (see Section 4 for details). The positions of mutated amino acid residues are indicated on the ribbon with the same color-code as in (**A**). PRP8_3H structure is superimposed on the corresponding structure of human ACE2 (ACE2-RBD crystal structure, PDB code 6M17), with the SARS-CoV-2 RBD surface shown in red. (**C**) Same as (**B**) for the WTL_3H (upper image) and PRP8_1H (lower image) ACE2 fragment polypeptide derivatives.

Two out of three V2.4 mutations [42] (T27Y, L79T, and N330Y), located on α-helix I and II (Figure 1A,B), were then introduced into our tri-helical ACE2 mini-protein with the aim of enhancing its affinity for the spike RBD while preserving its structural integrity. These amino acid replacements have previously been shown to enhance soluble ACE2 ectodomain binding to the RBD by about 40-fold [40,42] either directly, through the establishment of additional polar interactions, as in the case of Y27, or indirectly, by strengthening existing bonds, as for the 79T and 330Y substitutions [34]. An additional modification was the replacement of two hydrophobic leucine residues on the surface of α-helix III (not directly involved in RBD recognition) with glutamine residues in order to improve protein solubility [48]. The resulting tri-helical ACE2 mini-protein, designated as PRP8_3H, is 83 amino acids long, and its three-dimensional structure was successfully predicted with good accuracy (Figure 1B). PRP8_3H, which is predicted to be “non-allergenic” by Allertop v.2 [56], displays a minimal increase in the number of predicted antigenic determinants (from 3 to 4) and antigenic propensity (from 1.0026 to 1.0094) with respect to the corresponding wild-type ACE2 polypeptide fragment (see Section 4 for the prediction tools) [57]. 

We also designed two additional ACE2 fragments (Figure 1B and Table S1) to be used as reference polypeptides for the functional evaluation of PRP8_3H: (i) a wild-type-like tri-helical fragment, designated as WTL_3H, only containing the two Leu = >Gln substitutions in α-helix III (Figure 1C); and (ii) a monohelical N-terminal fragment harboring the eight P8 mutations and one v 2.4 mutation (T27Y), designated as PRP8_1H (Figure 1C).

### 2.2. Biochemical Properties of Recombinantly Expressed ACE2 Fragment Polypeptides

**Figure 2:**
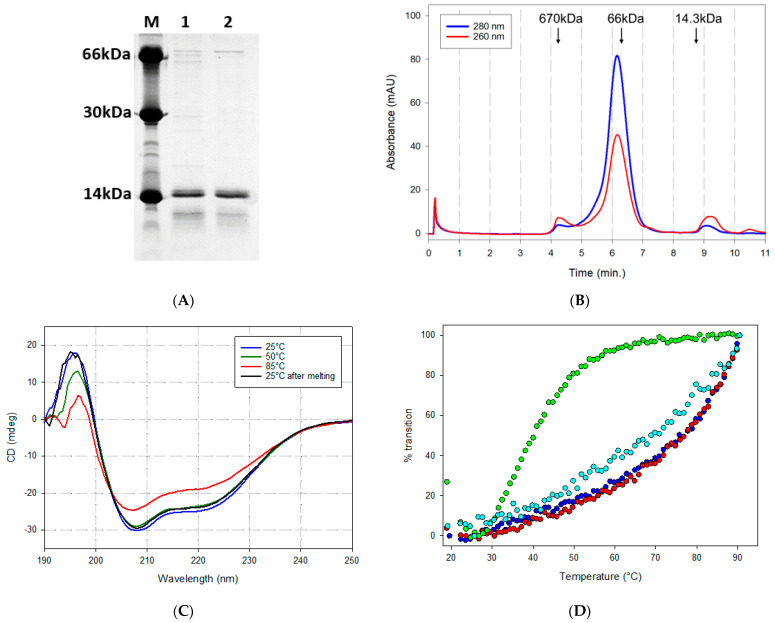
Biochemical characterization of the engineered PRP8_3H ACE2 polypeptide. (**A**) SDS-PAGE profile of the purified P8PE_3H protein after the metal-affinity chromatography (lane 1) and SEC (lane 2) purification steps (see Section 4 for details); the migration positions of molecular size standards (bovine serum albumin, carbonic anhydrase, and lysozyme) are shown in lane “M”. (**B**) SEC analysis of purified PRP8_3H (see Section 4 for details); the sizes and elution positions of molecular mass standards (thyroglobulin, bovine serum albumin, and lysozyme, from left to right) are indicated; blue line: absorbance at 280nm, red line: absorbance at 260 nm. (**C**) Far-UV circular dichroism spectra (190–250 nm) of PRP8_3H recorded at different temperature, as indicated. (**D**) Thermal stability of PRP8_3H (blue circles), PRP8_1H (red circles), WTL_3H (green circles), and the LCB1 mini protein (cyan circles) derived from CD spectrum transitions at 222 nm measured at temperatures ranging from 20 to 98 °C, as indicated.

The PRP8_3H fragment (14.3 kDa, pI = 5.06) was expressed in *E. coli* as an N-terminally His-tagged polypeptide also bearing a C-terminal c-myc tag (yield of ~40 mg/L of bacterial culture). It was purified to near homogeneity by metal-affinity chromatography (Figure S2A and Figure 2A), followed by an additional polishing step by size exclusion chromatography (SEC) (Figure S2B). The same expression and purification conditions were applied to the reference ACE2-derived polypeptides listed in Figure 1B, which included the mono-helical P8 peptide produced in recombinant form rather than by chemical synthesis [50] (see Figure S3A and Table S1, and Section 4 for details on the expression/purification procedures). PRP8_3H (expected MW = 14,300) eluted as a single symmetric peak upon SEC analysis, with an apparent molecular mass of 80 kDa (Figure 2B and Figure S2B). Under identical chromatographic conditions, PRP8_1H (expected MW = 6000) and WTL_3H (expected MW = 12,500) eluted with apparent molecular masses of approximately 60 kDa (Figure S3B) and 25 kDa (Figure S3C), respectively. Although a deviation toward higher apparent molecular weights is expected for elongated, non-globular proteins compared to globular proteins of the same size, SEC data clearly point to some kind of oligomerization that appears to be particularly prominent in the case of P8 substitutions-bearing polypeptides such as PRP8_3H and PRP8_1H. 

The conformation of PRP8_3H and its thermal stability were then investigated by circular dichroism (CD) spectroscopy. As shown in Figure 2C,D, the PRP8_3H far-UV CD spectrum is typical of an α-helical protein, with two minima at 208 and 222 nm and an estimated predominant (76%) α-helical secondary structure [58]. PRP8_1H also displays a CD spectrum typical of an α-helical polypeptide, as previously reported for the synthetic P8 peptide (Figure S3D). A CD spectrum typical of an α-helical protein was also observed, at room temperature, for the wild-type-like WTL_3H ACE2 polypeptide that lacks both the V2.4 and the stabilizing P8 mutations (Figure S3E). The thermal stability of PRP8_3H and of the two reference polypeptides was then examined by monitoring the change in ellipticity at 222 nm as a function of temperature (up to 90 °C). The results, reported in Figure 2D, evidenced some striking differences. In particular, an extremely strong thermal stability, with an estimated Tm > 95 °C, was observed for the PRP8_3H polypeptide, which was only partially denatured at 90 °C and, following a two-hour exposure to 100 °C, almost completely recovered its native structure upon incubation at 25 °C. A denaturation profile similar to that of PRP8_3H was observed for the mono-helical PRP8_1H polypeptide. In stark contrast with the extremely high thermal stability displayed by the P8 substitution-bearing polypeptides, the wild-type-like WTL_3H polypeptide, with an estimated Tm of approximately 39 °C, was found to be highly thermally unstable. These results clearly indicate the crucial role played by the P8 amino acid substitutions in the stabilization of the two PRP8 polypeptides regardless of their different molecular sizes (one or three helices). The thermal stability of PRP8_3H was also significantly higher than that of the LCB1 mini-protein decoy (see Figure 2D), whereas the wild-type-like WTL_3H polypeptide lacking the P8 modifications displayed a significant loss of secondary structure already at 37 °C, thus raising serious concerns regarding its possible utilization as an anti-SARS-CoV-2 decoy under physiological conditions. 

### 2.3. Spike RBD Binding by PRP8_3H and Other ACE2 Fragments 

**Figure 3:**
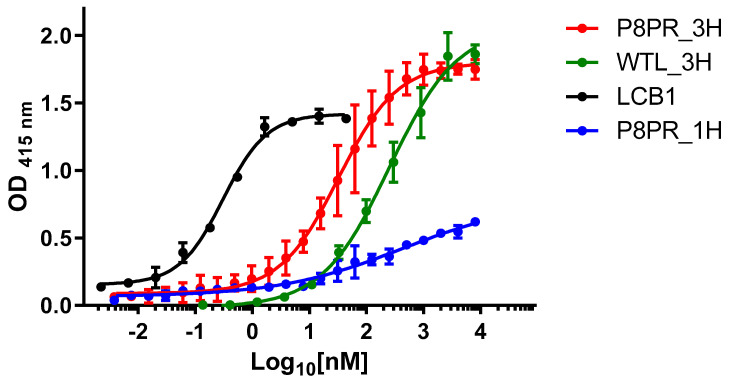
Binding of P8PR_3H, other ACE2 fragments, and the LCB1 artificial mini-protein to SARS-CoV-2 Wuhan spike RBD measured by ELISA. Concentration-dependent binding of P8PR_3H (red), WTL_3H (green), P8PR_1H (blue), and the LCB1 positive control (black) analyzed by ELISA, using Wuhan SARS-CoV-2 spike RBD as capture agent; data, expressed as maximum absorbance at 415 nm, are the mean of triplicate measurements (error bars represent the standard deviation). The EC50 estimated values are 32 nm, 253 nm, 420 nm, and 0.3 nM for P8PR_3H, WTL_3H, P8PR_1H, and LCB1, respectively.

The anti-SARS-CoV-2decoy capacity of the PRP8_3H ACE2 polypeptide was initially investigated with an ELISA performed at 37 °C using the Wuhan spike RBD as capture agent. As shown in Figure 3, PRP8_3H binds the RBD with an estimated EC50 of 32 nM. By comparison, the reference WTL_3H polypeptide bound the RBD with a median transition centered around 250 nM, while an EC50 of approximately 420 nM was estimated for the mono-helical PRP8_1H peptide, despite some technical difficulties and a non-saturating binding curve. Under identical experimental conditions, the artificial mini-protein LCB1 decoy displayed an expectedly higher potency, with an EC50 (0.3 nM) in the sub-nanomolar range.

We also calculated the apparent dissociation constants (Kd) of the best RBD binders, by fitting a Michaelis–Menten curve to the ELISA dose-response data [59] (Figure S4A,B and Table 1). The apparent Kd for the PRP8_3H polypeptide was 24 nM, while an approximately 10-fold lower value (0.2 nM), in good agreement with the Kd previously determined with a different experimental approach [54], was calculated for the LCB1 mini-protein decoy (Figure S4B and Table 1). The estimated Kd for the WTL_3H reference polypeptide was 192 nM, whereas the same analysis could not be applied to the PRP8_1H peptide due to its poor RBD binding profile.

We also performed non-competitive immunoassays [60], carried out in the presence of decreasing amounts of RBD, to determine the affinity constant (Kaff) of PRP8_3H (Figure S4C). A Kaff of 1.42 × 10^7^ M^−1^ was thus determined, which corresponds to an estimated Kd of 70.4 nM. Considering the different experimental procedures utilized for binding analyses, the latter value appears to be in keeping with the Kd determined by direct ELISA measurements (24 nM). Altogether, this indicates a 6- to 15-fold increased dissociation constant for RBD binding by PRP8_3H compared to the ~4 nM Kd previously reported for the entire ACE2 monomeric ectodomain bearing the whole set of v2.4 mutations [42]. The PRP8_3H polypeptide, with its strikingly high thermal stability, thus stands out among the examined ACE2 fragments as the best anti-SARS-CoV-2 decoy candidate.

### 2.4. Multivalent SARS-CoV 2 Pseudovirus Neutralization by the PRP8_3H Decoy 

**Figure 4:**
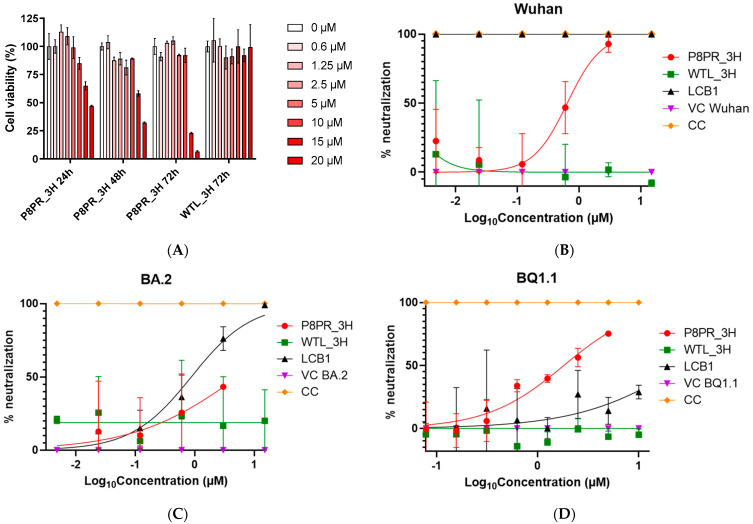
Cell viability and SARS-CoV-2 pseudovirion neutralization assays. (**A**) A cell viability assay was performed with the MTT dye on HEK293T cells incubated for different times at 37 °C with PRP8_3H or WTL_3H at the concentration indicated (from 0.6 to 20 μM; see Section 4 for details). As a reference, phosphate buffer was used instead of protein. Data, expressed as percent survival, relative to the ‘vehicle only’ control, were collected at the time indicated after treatment and represent the mean ± SD of three technical replicates. (**B**) Neutralization of SARS-CoV-2 spike Wuhan-Hu-1 pseudovirions by different engineered ACE2-derived fragments and the LCB1 mini-protein. (**C**) Same as (**B**) with Omicron BA.2 spike-expressing pseudovirions. (**D**) same as (**B**) with Omicron BQ 1.1 spike-expressing pseudovirions. CC (cell control, i.e., cells incubated without any added pseudovirus) and VC (virus control, i.e., cells incubated in the absence of any ACE2-mimicking polypeptide) represent the positive and negative controls, respectively; data are the mean (±SD) of two independent replicates (see Section 4 for details).

As a final step in the validation of the decoy activity of the engineered PRP8_3H ACE2 derivative, we tested its ability to neutralize SARS-CoV 2 pseudovirions. Preliminary viability assays, performed at different PRP8_3H concentrations and incubation times, were conducted in the HEK293T cell line utilized for neutralization assays. After an initial morphological examination, which did not reveal any detectable alteration in HEK293T cells exposed for 72 h to PRP8_3H up to a 10 µM concentration, we used the MTT assay to examine cell viability after a 72 h incubation at 37 °C. As shown in Figure 4A, a decrease in cell viability of less than 10% was detected in the presence of 5 µM PRP8_3H, a concentration ~200 times higher than its estimated Kd for the spike RBD. An approximately 10% decrease of cell viability was observed at a 10 µM PRP8_3H concentration after 72 h, while at 15 µM and 20 µM a significant reduction of cell viability was already evident after 24 h and became more pronounced at longer incubation times (Figure 4A). With a PRP8_3H preparation only processed by a metal affinity column (w/o SEC), inhibition progression was already evident at a 10 µM concentration, suggesting that a contaminant may contribute to this slight deterioration of cell viability. The WTL_3H native-like decoy is completely devoid of toxicity under the same experimental setup up to 20 µM after 72 h incubation (Figure 4A). 

Neutralization assays were initially performed against Wuhan SARS-CoV-2 pseudotyped lentivirus particles. As shown in Figure 4B, PRP8_3H blocked the infection of HEK293T cells with a half-inhibitory concentration in the sub-micromolar range (IC_50_: 0.65 µM). No inhibitory effect could be detected with the WTL_3H (wild-type-like, non-P8) polypeptide, even at a 15 µM concentration, likely due to its extreme thermal instability. As expected, under the same experimental conditions, a potent neutralizing activity in the low nanomolar range was observed with the LCB1 mini-protein (Table S2). 

Similar neutralization assays were performed against Omicron SARS-CoV-2 BA.2 and its more recent BQ.1.1 variant [61], as summarized in Supplementary Table S2. PRP8_3H neutralized Omicron BA.2 spike pseudovirions at micromolar (IC_50_: 5.30 µM) concentrations (Figure 4C) and was even more effective against the BQ 1.1 Omicron variant (IC_50_: 1.81 µM; Figure 4D). Again, no neutralization effect could be detected with the thermolabile WTL_3H polypeptide. At variance with PRP8_3H, the LCB1 mini-protein, as previously reported [53], displayed a marked drop in neutralizing activity against Omicron spike-bearing pseudovirions, which was particularly prominent in the case of the BQ.1.1 variant (Figure 4D). The PRP8_3H neutralization data highlight the validity of an ACE2-derived approach design, which targets the invariable region of the spike protein involved in ACE2 interaction, potentially overcoming the challenge of viral escape mutations, which instead are severely affecting the neutralization capability of artificial mini-protein LCB1.

## 3. Discussion

The mutagenic drift of SARS-CoV-2 and the continuing appearance of novel, vaccine immunity-escaping VOCs has prompted the search for broad coverage treatments. These include a few pan-vaccine prototypes and broadly neutralizing mAbs targeting highly constrained, and thus less prone to variation, spike epitopes mainly located on the S2 domain [62,63,64,65]. An alternative, initially overlooked, approach exploits the invariant spike region interacting with the ACE2 cellular receptor, which is essential for virus internalization, for the construction of natural or artificial viral blocking decoys. The therapeutic and prophylactic efficacy of the ACE2 ectodomain and engineered fragments thereof has been demonstrated against a number of SARS-CoV-2 variants, including some of the most recent Omicron sub-lineages [66,67,68]. 

The aim of the present study was to gain a more detailed, comparative insight into the stability, RBD binding, and virus neutralization capacity of different engineered ACE2 fragments, focusing particularly on PRP8_3H, a newly designed three-helical ACE2 derivative. Our data confirmed the conformational stability issues posed by ACE2 fragments with an elongated α-helix structure. A typical example, in this regard, is the wild-type-like, N-terminal ACE2 fragment WTL_3H, which was confirmed to be extremely thermolabile [48], with a nearly complete loss of native secondary structure at 37 °C, and thus devoid of any detectable neutralizing activity under our experimental conditions. We also confirmed that α-helix, I-containing, ACE2-derived polypeptides (even those comprising three N-terminal ACE2 α-helices) need to be modified in order to maintain their native conformation. The substitution of a few α-helix I amino acids not engaged in the spike RBD interaction with aliphatic residues, as in the P8 peptides, has been shown to stabilize the peptide conformation while preserving its SARS-CoV-2 neutralization properties [50]. We verified the enhanced thermal stability of a recombinantly expressed, properly folded mono-helical P8 peptide also harboring one (T27Y) of the three V2.4 mutations [42]. The recombinant product (PRP8_1H), however, was found to be a rather poor RBD binder, with an estimated binding affinity significantly lower than that previously reported for the chemically synthesized P8 peptide lacking the T27Y substitution. This unexpected discrepancy might be due to the different production mode, including the possible incorporation in the recombinant peptide of RBD binding-interfering post-translational modifications, and/or to an unpredictably negative effect of the T27Y substitution. What is clear, however, is that decoys only relying on helix-1, while reproducing a number of ACE2-RBD contacts, are missing key interacting residues located on α-helix-II. In fact, various in vitro deep mutational scanning experiments, as well as in silico studies performed on ACE2, identified a number of RBD affinity-enhancing amino acid substitutions, a subset of which is located on α-helix-II [17,39,40,42,68]. In addition to a better intrinsic conformational stability, three-helix decoys can accommodate more RBD-interacting residues, thus expanding the range of amino acid residues whose mutation could improve decoy potency. 

For these reasons, the main focus of our work was to design, recombinantly produce, and test an engineered three-helical, N-terminal, ACE2 derivative (PRP8_3H) combining the stabilizing effects of α-helix-I P8 mutations [50] with the previously reported V2.4 RBD affinity-enhancing amino acid substitutions on α-helix-I and II [42]. In particular, PRP8_3H harbors eight P8 stabilizing and two V2.4 substitutions, which led to a fully structured three-helix fragment polypeptide with a remarkable thermal resilience and a broad-range SARS-CoV-2 pseudovirus neutralization capacity. Compared to full-length ACE2 ectodomain-based decoys, ACE2-derived fragments can reach higher local concentrations at the neutralization site and also allow for easier manufacturing. Regarding thermal stability, CD data indicate that the P8 substitutions are required to achieve a strongly enhanced structural stabilization, compared, for example, to the wild-type-like WTL_H3 polypeptide. Thermal resilience, together with high-yield production in a bacterial system, make PRP8_3H an excellent candidate for different pharmaceutical applications, including dry-powder prophylactic inhalable formulations produced by high-temperature spray-drying procedures [69]. 

As revealed by SEC analysis, a likely side-effect of P8 aliphatic residue substitutions was a tendency toward the formation of multimeric aggregates. These were particularly evident in the case of PRP8_3H, which featured a six-fold increase in the apparent vs. the expected molecular weight, compared to an only two-fold increase for the non-P8 WTL_3H polypeptide. Such an increase is too large to be simply accounted for by the non-globular nature of the PRP8_3H polypeptide. Interestingly, however, both PRP8_3H and the recombinantly expressed mono-helical P8 peptide (10-fold increase in apparent MW) eluted from size exclusion chromatography as relatively sharp, symmetric peaks, suggesting the formation of well-defined multimeric species rather than disordered random aggregates.

Some cellular toxicity of the PRP8_3H polypeptide, especially at high concentrations, was revealed by MTT assays. The fact that no toxicity was observed with the WTL_3H polypeptide suggests that such an effect might at least in part be related to the aliphatic P8 substitutions. It should also be noted, however, that PRP8_3H toxicity was significantly lower in the SEC-purified polypeptide compared to the polypeptide only subjected to the metal affinity chromatography purification step. An alternative, not mutually exclusive, possibility is thus that hard-to-remove contaminants may contribute to PRP8_3H toxicity, an effect that might be eliminated (or at least reduced) with the use of more effective manufacturing/purification procedures and/or through recombinant expression in a different cellular system. 

PRP8_3H, which contains two out of three RBD affinity-enhancing V2.4 mutations, featured a nanomolar Kd for the Wuhan spike RBD. This is comparable to the Kd value previously reported for the wild-type, full-length human ACE2 ectodomain in monomeric form [42], but it is 6- to 15-fold higher than that of an ACE2 ectodomain derivative bearing the entire set of V2.4 mutations [42].

While the V2.4 mutations at positions 27 and 79 can act either directly by forming new contacts, or indirectly by changing the conformations of adjacent residues, the lower estimated affinity of PRP8_3H could in principle be ascribed to the missing V2.4 N330Y mutation. This involves an amino acid residue lying outside of the PRP8_3H sequence, thus precluding a complete reconstitution of all the intermolecular contacts present at the ACE2-RBD interface. One could also imagine, however, that the eight P8 substitutions present in PRP8_3H, while strikingly improving conformational and thermal stability, may negatively affect backbone flexibility and physiological helix I–II bending, ultimately causing a reduction in RBD affinity with respect to the full-length ACE2 V2.4 protein. Similar considerations may explain the reduced neutralizing activity of PRP8_3H compared to the entire ACE2 ectodomain harboring the whole set of V2.4 mutations. 

Despite the above-described limitations, the ACE2-derived PRP8_3H decoy represents a promising prototype in protein-based therapeutics against SARS-CoV-2, demonstrating the potential of rational design strategy in creating stable and effective therapeutic candidates with enhanced structural stability. 

The engineered PRP8_3H polypeptide combines broad neutralization capacity at (sub)micromolar concentrations against multiple SARS-CoV-2 variants, with enhanced thermostability at physiological temperatures, addressing the key limitations of previous protein-based therapeutics. Moreover, the PRP8_3H polypeptide ability to target the conserved ACE2-binding interface, coupled with minimal sequence modifications, suggests both reduced likelihood of escape mutations and limited immunogenicity concerns. 

Further studies will also take advantage of PRP8_3H as a reference polypeptide for in vitro deep scanning mutagenesis experiments aimed at identifying a further expanded set of RBD-binding-enhancing mutations to validate long-term efficacy against emerging variants. Other experiments will explore the effect of targeted decoy multimerization, which has proven to be particularly effective for increasing the potency of other ACE2 decoys [42,53]. To fully exploit the PRP8_3H therapeutic potential, the development of respirable dry-powder formulations and investigation of intranasal delivery methods could expand its clinical applications, while optimization of production processes and formulation conditions will be crucial for successful clinical translation. 

PRP8_3H thus lends itself as a promising prototype for the development of even more potent decoy derivatives, to be used in different physico-chemical formulations, as a valuable alternative to monoclonal antibodies for a mutational escape-insensitive prophylactic approach against SARS-CoV-2. Future research will expand these findings to advance our understanding of protein engineering and our capability to develop effective antiviral therapeutics. 

## 4. Materials and Methods

### 4.1. Structure Predictions

The SWISS-MODEL 3D structure prediction server was used for ACE2 fragment structure prediction. The structure of PRP8_3H (QMEANDisCo Global score: 0.81 ± 0.09) was built using the crystal structure of the human ACE2 mutant protein bound to the Gamma spike RBD (PDB: 7WNM) as a template. The same structure was employed as a template for the structure prediction of PRP8_1H (QMEANDisCo Global score: 0.72 ± 0.12). The structure of WTL_3H was predicted using human ACE2 bound to Delta RBD (PDB: 7W9I, QMEANDisCo Global score: 0.83 ± 0.09) as a template. 

### 4.2. Expression and Purification of ACE2-Derived Polypeptide Fragments

Codon-optimized sequence coding for c-myc tagged ACE2 polypeptide fragments were chemically synthesized (GenScript Biotech Corp., Piscataway, NJ, USA) (see Supplementary Table S1). PRP8_3H, PRP8_1H, and LCB1 coding sequences were inserted into the NcoI and XhoI sites of a 6xHis-tag pET28a(+) plasmid (MilliporeSigma, Merck KGaA, Darmstadt, Germany)), while WTL_3H was inserted into the NdeI and XhoI sites of a 6xHis-tag pET26b(+) plasmid (MilliporeSigma, Merck KGaA, Darmstadt, Germany). The resulting constructs were transformed into *Escherichia coli* BL21 codon plus (DE3)-RIL cells (Agilent Technologies, Santa Clara, CA, USA, catalogue number 230,245) for recombinant protein expression. P8PR_3H and P8PR_1H were induced overnight at 20 °C in LB medium supplemented with 1 mM isopropyl-β-D-thiogalactopyranoside (IPTG); WTL_3H and LCB1 were induced at 20 °C for 24 h under auto-induction conditions (Auto Induction Medium, AIM-LB broth base; Formedium Ltd., Hunstanton, Norfolk, United Kingdom). Cells were harvested by centrifugation at 9000× *g* for 10 min and lysed by sonication (Misonix Sonicator 3000) in 50 mL of 25 mM Tris-HCl (pH 7.5) and 0.3 M NaCl (buffer A) supplemented with Protease Inhibitor Cocktail (Sigma-Aldrich Corporation, St. Louis, MO, USA). After an additional centrifugation (15,000× *g* for 30 min at 4 °C), the soluble supernatant fraction derived from a 1 L bacterial culture (typically a 50 mL volume) was immediately subjected to metal-affinity chromatography on a HisTrap FF crude column (5 mL; Cytiva (formerly GE Healthcare Life Sciences), Marlborough, MA, USA) pre-equilibrated in Buffer A containing 20 mM imidazole at 2.5 mL/min. After extensive washing, bound polypeptides were eluted by applying an 18 min linear gradient of buffer A containing 500 mM imidazole. After SDS-polyacrylamide gel electrophoresis (SDS-PAGE) analysis, protein-containing fractions were pooled, concentrated, and loaded onto a size exclusion Superdex 200 HR10/30 column (24 mL; GE Healthcare Chicago, IL, USA) equilibrated in Buffer A. Elution was performed at a flow-rate of 0.5 mL/min using an ÄKTA Pure 25 M (GE Healthcare, Chicago, IL, USA) chromatographic system; individual protein-containing fractions were analyzed by SDS-PAGE, pooled, and stored at −80 °C. A calibration curve was built with the use of thyroglobulin (670 kDa), bovine serum albumin (66 kDa), ovalbumin (44.3 kDa), trypsinogen (24.5 kDa), and lysozyme (14.5 kDa) as molecular mass standards. The same purification protocol was applied to all the ACE2 polypeptides examined in this work, except for the mono-helical PRP8_1H polypeptide. In this case, to overcome possible technical problems caused by the small size of PRP8_1H, we designed a dimer polypeptide comprising two PRP8_1H units spaced by a flexible linker with an internal TEV proteolytic site (Table S1). To release PRP8_1H, the metal-affinity purified dimeric polypeptide was incubated for 15 h at 4 °C with an N-terminally His-tagged TEV protease derivative (1:50 *w*/*w*). Following imidazole removal by diafiltration, the TEV-treated product was subjected to an additional metal-affinity chromatography in order to capture the His-tagged TEV protease, while collecting the cleaved monomeric PRP8_1H polypeptide lacking an N-terminal His-tag but still containing the C-terminal c-myc tag. After SDS-PAGE analysis, PRP8_1H-containing fractions were pooled, concentrated, and stored at −80 °C.

The pcDNA3-SARS-CoV-2-S-RBD-8his plasmid [42] (a kind gift from Erik Procko; Addgene plasmid # 145145; http://n2t.net/addgene:145145; RRID:Addgene_145145) was used for His-tagged RBD expression in HEK293T cells (ATCC: CRL-11268), which were first seeded into 182 cm^2^ flasks (ET7181 Primo^®^ TC Flask 182 cm^2^ screw cap w/filter, Euroclone; 4.9 × 106 cells/flask) in Dulbecco’s modified Eagle’s medium (DMEM, Gibco, Thermo Fisher Scientific, Waltham, MA, USA) containing 10% fetal bovine serum (FBS, Gibco) and incubated at 37 °C with 5% CO_2_. After 24 h, the medium was removed and replaced with DMEM/F12 1:1 (Cytiva (formerly GE Healthcare Life Sciences), Marlborough, MA, USA) without FBS, and the cells were transfected with pcDNA3-SARS-CoV-2-S-RBD-8his using PEI MAX^®^-Transfection Grade Linear Polyethylenimine Hydrochloride (MW 40,000) reagent (Polysciences, Inc., Warrington, PA, USA). To this end, 50 μg of plasmid DNA were mixed with 150 μg of PEI in 5 mL of Opti-MEM™ I (Gibco) (DNA:PEI, 1:3 ratio), and after 15 min of incubation at room temperature the mixture was added drop-wise to the cells. Three days post-transfection, the medium was collected, centrifuged at 9000× *g* for 15 min, and, after adjusting the pH to 7.8, loaded onto a 5 mL HisTrap excel column (Cytiva (formerly GE Healthcare Life Sciences), Marlborough, MA, USA) pre-equilibrated in buffer A at a flow of 2.5 mL/min. The His-tagged RBD protein was eluted by applying a 15 min linear gradient of buffer A containing 500 mM imidazole. Following SDS-PAGE analysis, pooled and concentrated protein fractions were subjected to size exclusion chromatography (Superdex 200 increase, 5/150 GL column). The purified RBD, which eluted as a monomer, was exchanged into 25 mM Tris-HCl (pH 7.75), 0.15 M NaCl, and stored at −80 °C. 

### 4.3. Analytical Size Exclusion Chromatography

The apparent native molecular weights of the ACE2 fragment polypeptides were estimated by size exclusion chromatography (SEC). This was performed at a 0.3 mL/min flow rate using an AKTA pure 25 M Chromatographic system (GE Healthcare, Chicago, IL, USA) and a Superdex 200 increase 5/150 GL column (3 mL; GE Healthcare, Chicago, IL, USA) equilibrated in 100 mM potassium phosphate buffer (pH 7.5), 150 mM NaCl. Thyroglobulin (670 kDa), bovine serum albumin (66 kDa), ovalbumin (45 kDa), and lysozyme (14.5 kDa) were utilized as molecular mass standards for column calibration.

### 4.4. Far-UV Circular Dichroism

Far-UV CD spectra (190–250 nm) were acquired at 25 °C on 15 µM polypeptide solutions in 10 mM phosphate buffer (pH 7.4) using a Jasco J1500 Spectropolarimeter equipped with a Peltier thermostated temperature controller. Each spectrum was the average of three accumulations recorded using a 0.2 cm path-length cuvette, a bandwidth of 1 nm, a data interval of 0.5 nm, D.I.T of 2 s, and a scanning speed of 50 nm/min.

Analysis/deconvolution of secondary structure from CD spectra was performed on the K2D3 server, using a neural network-based method [58]. CD spectra were analyzed from 195 to 240 nm; CD and K2D3 output was within the quality fit threshold value.

Thermal denaturation analysis was performed at 222 nm (D.I.T: 8 s; bandwidth: 1.00 nm) on 15 µM polypeptide samples using the variable temperature program in the 20–90 °C temperature range, with a ramp rate of 5 °C/min.

### 4.5. ELISA

For ELISA, 50 µL of His-tagged RBD, diluted to a final concentration of 250 nM in phosphate buffered saline (PBS), were added to the wells of microtiter plates (SpectraPlate-96 HB; Perkin Elmer) and incubated overnight at 4 °C. The wells were then washed three times with 300 μL of PBS containing 0.2% (*v*/*v*) Tween-20 (PBS-T) and blocked at 37 °C for 1 h with 200 μL of 3% (*w*/*v*) skimmed milk (Sigma-Aldrich) dissolved in PBS-T, followed by three additional washings with PBS-T. Two-fold serial dilutions (starting from an 8 µM concentration) of P8PR_3H or P8PR_1H and three-fold serial dilutions of WTL_3H (starting from 8 µM) or LCB1 (starting from 44 nM) were added to the wells and incubated at 37 °C for 1 h. After washing three times with 300 μL of PBS-T, 100 μL of a goat anti-myc tag HRP conjugated antibody (A190-104P; Bethyl Laboratories, Inc., Montgomery, TX, USA) diluted 1:1000 in PBS were added, and the plates were incubated at 37 °C for 1 h. After three additional washes with PBS-T, 100 μL of 2,2′-azino-bis (3-ethylbenzthiazoline-6-sulfonic acid) peroxidase substrate (ABTS; SeraCare Life Sciences, Milford, MA, USA) were added to each well, and after a 30 min incubation at 30 °C the signal produced by ABTS oxidation was determined by measuring the absorbance at 415 nm with a microplate reader (iMark™ Microplate Absorbance Reader; Bio-Rad, Hercules, CA, USA); all measurements were conducted in triplicate (*n* = 3). ELISA dose-response data were fitted by a nonlinear log(agonist) vs. response-variable slope to determine EC50 and by a nonlinear Michaelis–Menten curve to determine apparent Kd values for the binding of each polypeptide to the RBD.

To determine the RBD affinity constant (Kaff) of P8PR_3H, we used an ELISA set-up previously described by Beatty et al. and Zhou et al. [60,69]. Briefly, plates were coated with different concentrations of His-tagged RBD (250 nM, 125 nM, 62.5 nM, and 31.25 nM) and, following incubation overnight at 4 °C and blocking at 37 °C for 1 h with 3% (*w*/*v*) skimmed milk, two-fold serial dilutions (starting from an 8 µM concentration) of P8PR_3H were added to the wells and incubated at 37 °C for 1 h. The HRP-conjugated anti-myc tag secondary antibody, diluted as above, was then added and, following incubation at 37 °C for 1 h, the ABTS substrate was added and absorbance was measured at 415 nm.

The Kaff was calculated as follows [60,70]:

$$n=\frac{\left[RBD\right]}{\left[{RBD}^{\prime }\right]}\;\;\;\;\;\;\;\;\;\;\;\;\;\;\;Kaff=\frac{n-1}{n\left[{P8PR\_3E}^{\prime }\right]-\left[P8PR\_3E\right]}$$

[P8PR_3H] and [P8PR_3H′] are the concentrations of the decoy corresponding to a 50% ODmax for plates coated with RBD concentrations of [RBD] and [RBD′], respectively.

### 4.6. Cell Viability Assays

PRP8_3H and WTL_3H were subjected to cell viability assays on confluent HEK293T cells, which were seeded in 96-well plates (100 μL/well) in DMEM medium containing 10% FBS at a density of 3 × 10^4^ cells/well and grown for 24 h at 37 °C in a 5% CO_2_ atmosphere. The medium was then removed and cells were treated with 100 μL of fresh medium containing increasing concentrations of PRP8_3H or WTL_3H (from 0.6 to 20 μM) for 24 h, 48 h, and 72 h, using cells treated with a buffer-only solution as controls. Then, 10 μL of the 3-(4,5-dimethylthiazol-2-yl)-2,5-diphenyltetrazoliumbromide (MTT) reagent (final concentration 0.5 mg/mL) were added to each well, and the plate was incubated for 4 h at 37 °C before the addition of 100 μL of the solubilization solution (10% SDS in 0.01 M HCl). After a 16 h incubation at 37 °C in a 5% CO_2_ atmosphere, reduction of MTT by viable cells and formation of the purple-colored formazan product was determined by measuring absorbance at 570 nm with a Varioskan™ LUX (Thermo Fisher Scientific, Waltham, MA, USA) microplate reader. Three technical replicates were performed for each ACE2-derived polypeptide. 

### 4.7. Neutralization Assays

Neutralizing activity against Wuhan, Omicron BA.2, and Omicron BQ 1.1 SARS-CoV-2 variants was determined using spike-displaying virus-like particles (VLPs; Integral Molecular Inc. Philadelphia, PA, USA) as well as pseudotyped lentiviral particles (VectorBuilder GmbH, Chicago, IL, USA), harboring, respectively, a renilla luciferase and a firefly luciferase reporter gene. The infection of engineered 293T cells (CoronaAssay-293T(hACE2-hTMPRSS2); VectorBuilder GmbH, Chicago, IL, USA) induces luciferase expression, which generates a light signal upon substrate supplementation: coelenterazine or luciferin, for renilla or firefly luciferase, respectively. Decoy-mediated neutralization of both particles thus results in a reduction of the light signal, measured as relative light units (RLU). 

Prior to neutralization assays, 293T hACE2-hTMPRSS2 cells suspended in DMEM-10 mM HEPES supplemented with 10% FBS and 1% Pen/Strep were seeded in 96-well microtiter plates at a density of 2 × 10^5^ cells/mL and incubated at 37 °C in a 5% CO_2_ atmosphere. The neutralization capacity of P8PR_3H, WLT_3H, and LCB1 was first assessed against Wuhan and Omicron BA.2 spike-displaying VLPs. To this end, Wuhan and Omicron BA.2 VLPs were first incubated for 1 h at 37 °C with five-fold serial dilutions (starting from a 15 µM concentration) of each of the three polypeptides (1:1 volume ratio). Each mix was then added to the plate-attached cells, followed by a 72 h incubation at 37 °C in a 5% CO_2_ atmosphere.

P8PR_3H, WLT_3H, and LCB1 were also tested at concentrations ranging from 0.078 to 10 µM, for neutralizing activity against Omicron BQ 1.1 variant spike-displaying lentiviral particles diluted 1:2560 in DMEM medium, as described above. Polypeptides were first incubated for 1 h at 37 °C with the spike-displaying lentiviral particles (1:1 volume ratio) in 96-well microtiter plates. 

Subsequently, the neutralization mix consisting of lentiviral particles and polypeptides was added to an equal volume of 293T hACE2-hTMPRSS2 cells suspension at a density of 2 × 105 cells/mL. The content of each well was rapidly transferred to a clear-bottom 384-well plate (Corning Life Science Plastic) with an Assist Plus liquid handler (Integra Biosciences) and incubated for 72 h at 37 °C. 

After incubation, cells exposed to each of the tested variants were lysed with Lysis Juice (PJK Biotech, Kleinblittersdorf, Germany) diluted 1:2 in cell-culture grade water and kept on a plate shaker (200 rpm) for 30 min at room temperature. Following automated injection of the renilla luciferase substrate coelenterazine (PJK Biotech, Germany) for the Wuhan and BA.2 spike-displaying VLPs, or the firefly luciferase D-Luciferin substrate (Beetle Juice; PJK Biotech, Germany) for BQ1.1 spike pseudotyped lentiviruses, plates were read at 560 nm with the multimode plate reader Mitras LB 940 (Berthold Technologies, Bad Wildbad, Baden-Württemberg, Germany). The RLU value of each tested sample was normalized with respect to the value measured in the cell control (CC), i.e., cells incubated in the absence of any pseudovirion (CC = 100% neutralization) and the virus control (VC), i.e., cells exposed to the pseudovirions in the absence of any neutralizing agent (VC = 0% neutralization).

## Figures and Tables

**Table 1:** Apparent Kd values for the binding of different ACE2 fragments and the LCB1 mini-protein to the SARS-CoV-2 Wuhan-1 spike RBD determined by ELISA.

Polypeptides	Kd ± SD (nM) *
P8PR_3H	24.3 ± 2.6
WTL_3H	191.8 + 21.5
LCB1	0.21 + 0.03

* Apparent Kd values + SD were calculated from ELISA dose–response data (reported in Figure 3), fitted to a Michaelis–Menten curve.

## Data Availability

The raw data supporting the conclusions of this article will be made available by the authors on request.

## Supplementary Materials

The following supporting information can be downloaded at: https://www.mdpi.com/article/10.3390/ijms252212319/s1.
