# Supplementary material for: Broad Neutralization Capacity of an Engineered Thermostable Three-Helix Angiotensin-Converting Enzyme 2 Polypeptide Targeting the Receptor-Binding Domain of SARS-CoV-2
Source: Int J Mol Sci. 2024 Nov 16;25(22):12319. doi: 10.3390/ijms252212319 (PMC11594380; doi:10.3390/ijms252212319)
Supplement: Supplementary file 1 [file ijms-25-12319-s001.zip › ijms-3288760-supplementary.pdf]

Figure S1

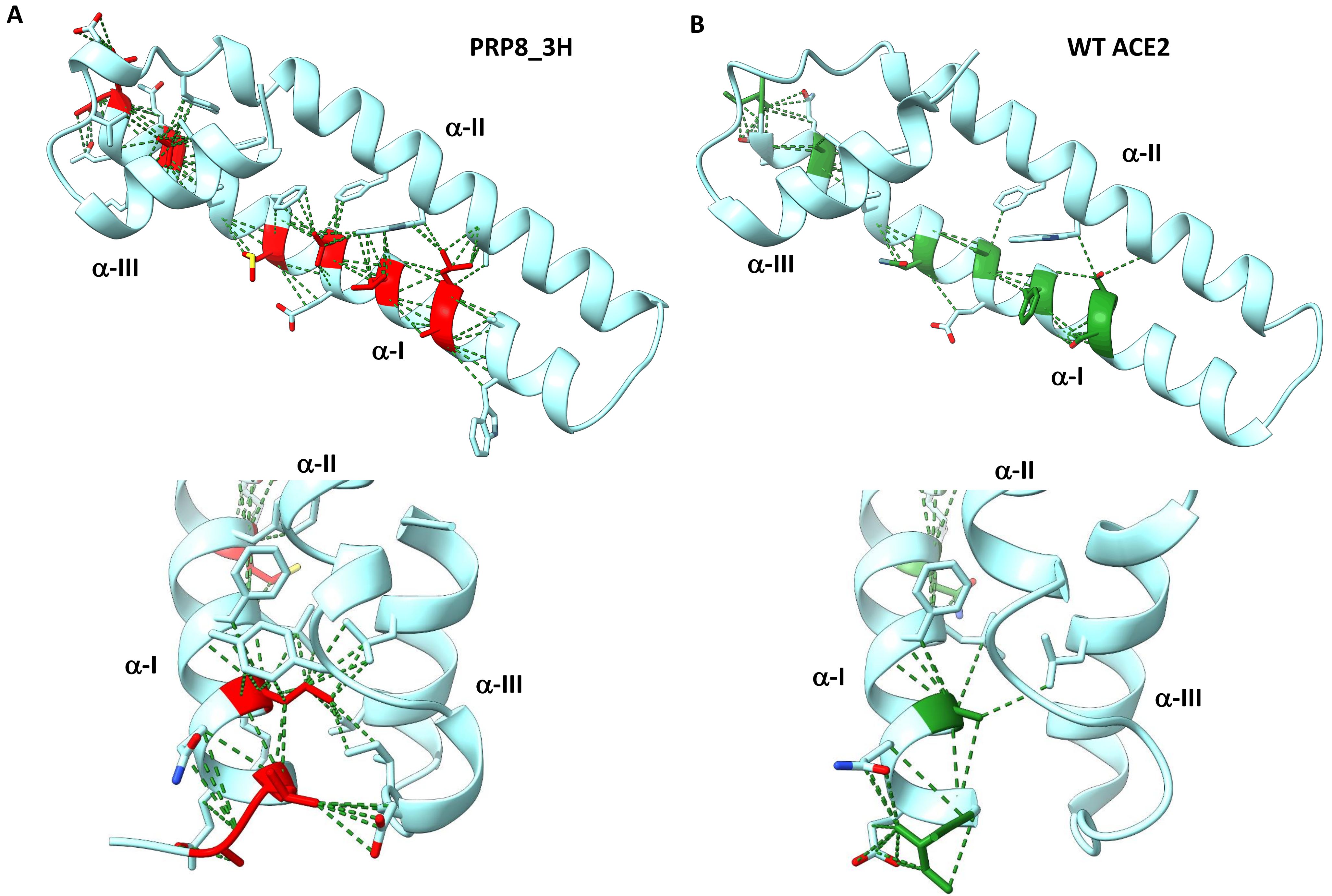
A
PRP8_3H
α-II
α-III
α-I
α-II
α-I
α-III
B
WT ACE2
α-II
α-III
α-I
α-II
α-I
α-III

Figure S1

Intramolecular contacts of PRP8_3H and wild type human ACE2 fragments **A.** Predicted structure of PRP8_3H (*light blue*) with stabilizing P8 mutations shown in *red*. The P8 mutated residues forming intramolecular contacts are displayed as *red* heteroatom colored sticks, with the target residues shown as *light blue* heteroatom; pseudobonds are shown as dotted *green* lines. Upper image: overall fragment; lower image: close view of the N- and C-terminal ends of PRP8_3H with α-III. **B.** Corresponding N-terminal region of wild-type hACE2 (PDB code: 6M17) with the amino acid residues targeted by the P8 substitutions present in PRP8_3H evidenced as *green* heteroatom colored sticks. Except for the *green* instead of *the* red colors, the color code is the same as in (**A**).

Figure S2

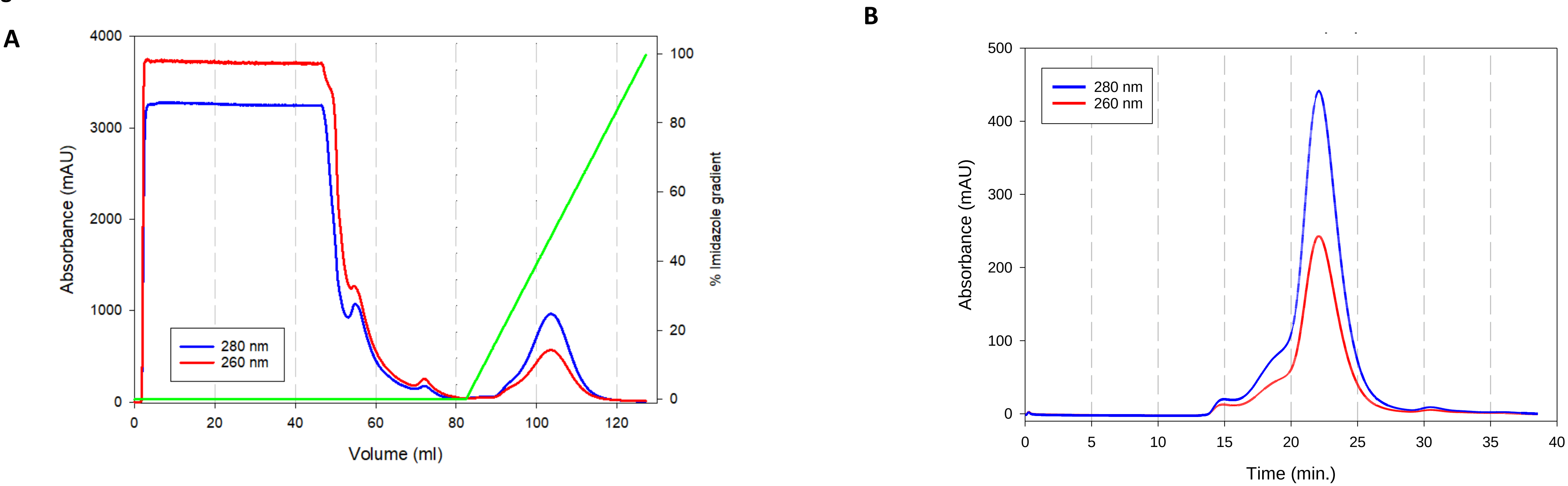

Figure S2. Purification and cytotoxicity analysis of the PRP8_3H ACE2 fragment. **A.** Representative metal-affinity chromatography (HisTrap FF Crude FF 5ml column) profile. **B.** Semi-preparative SEC (Superdex 200 10/300). **C.** Viability MTT assays performed on HEK293T cells incubated at 37°C with PRP8_3H or WTL_3H for different times at the indicated concentrations (see 'Materials and Methods' for details); phosphate buffer w/o any added protein (0 µM) was used as a baseline reference. Data, expressed as percent survival relative to the 'vehicle only' control, are the mean ± SD of three technical replicates.

Figure S3

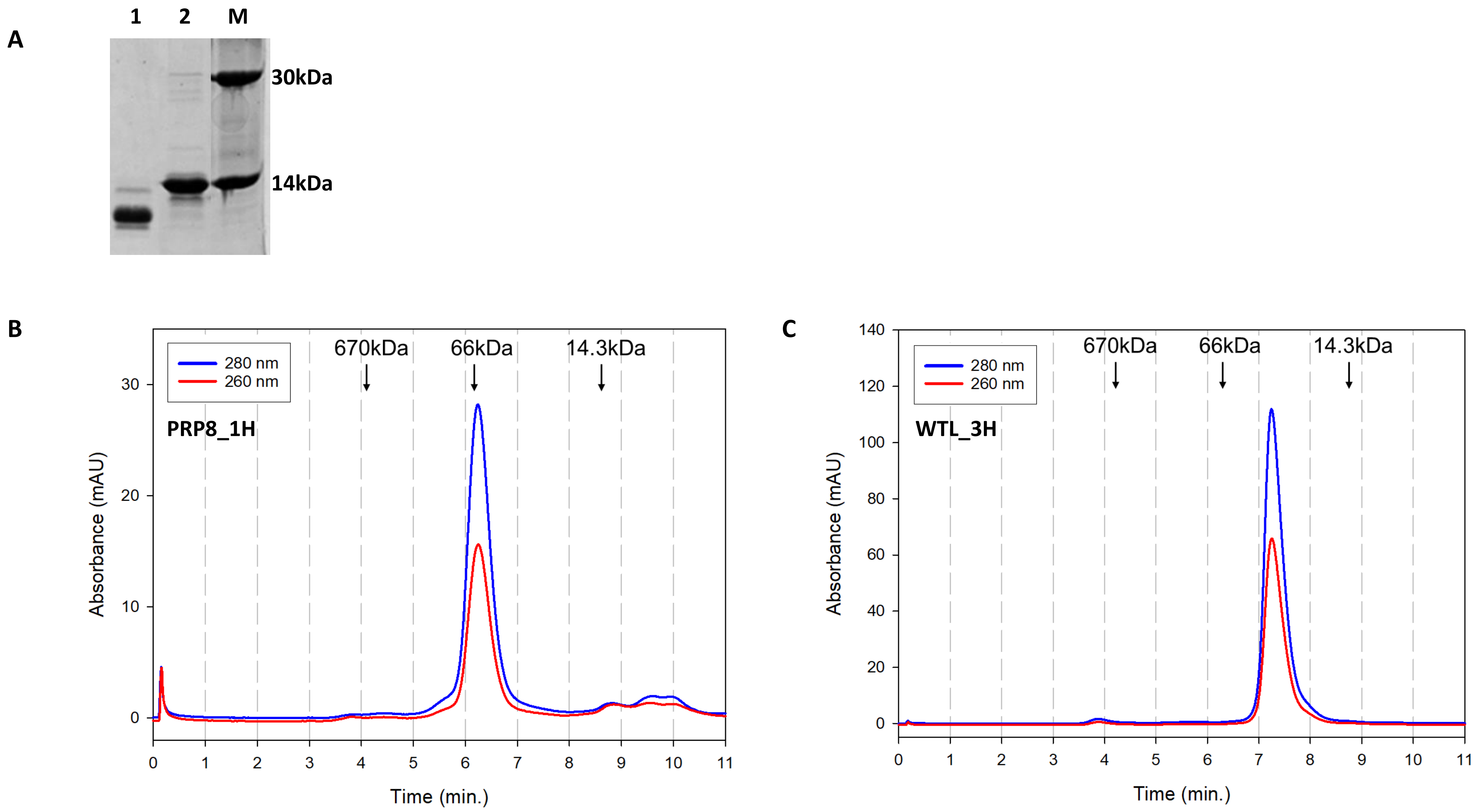
A
1
2
M
30kDa
14kDa
B
280 nm
260 nm
670kDa
66kDa
14.3kDa
PRP8_1H
Absorbance (mAU)
Time (min.)
C
280 nm
260 nm
670kDa
66kDa
14.3kDa
WTL_3H
Absorbance (mAU)
Time (min.)

Figure S3

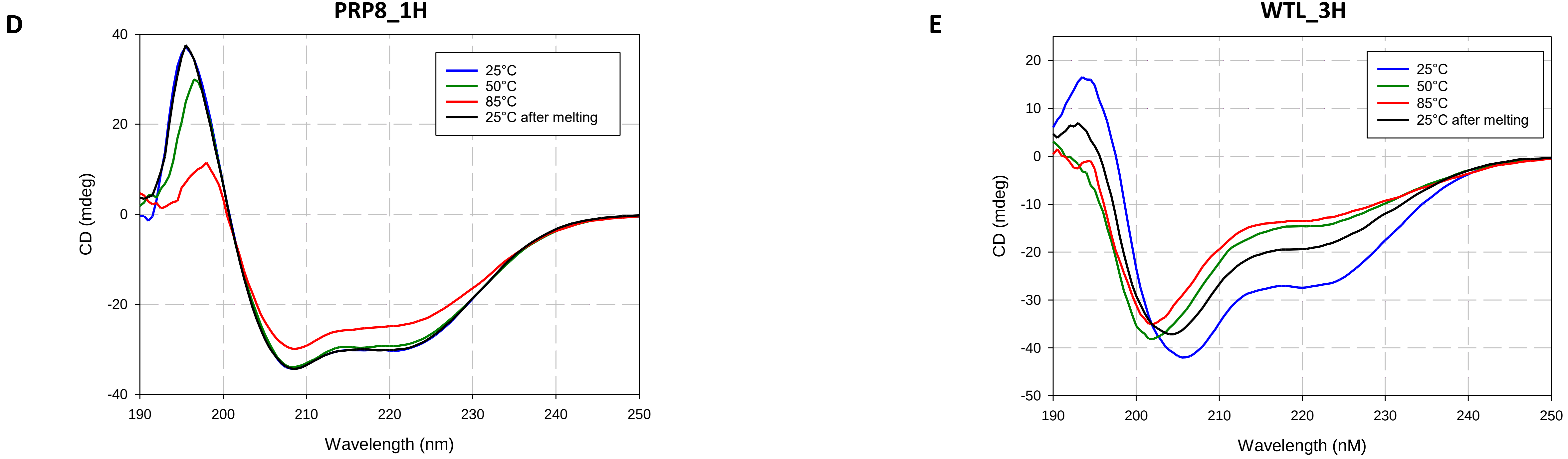

Figure S3. Biochemical characterization of the purified PRP8_1H and WTL_3H ACE2 fragments. **A.** SDS-PAGE analysis of the PRP8_1H (*lane 1*) and WTL_3H (*lane 2*) polypeptides; molecular mass markers (carbonic anhydrase and lysozyme) are shown in lane "*M*". **B.** SEC analysis (Superdex 200 Increase 5/150 GL column) of the purified PRP8_1H polypeptide. The sizes and elution positions of the molecular mass standards (thyroglobulin, bovine serum albumin and lysozyme, from *left* to *right*) utilized for column calibration are indicated by arrows; *blue line*: absorbance at 280nm, *red line*: absorbance at 260nm. **C.** same as (**B**) for the WTL_3H polypeptide. **D.** Far-UV circular dichroism spectra of the PRP8_1H polypeptide (15 µM) recorded between 190 and 250 nm at the indicated temperatures; also shown is the spectrum of the thermally denatured polypeptide after incubation at 25°C. **E.** Same as **(D)** for the WTL_3H polypeptide.

Figure S4

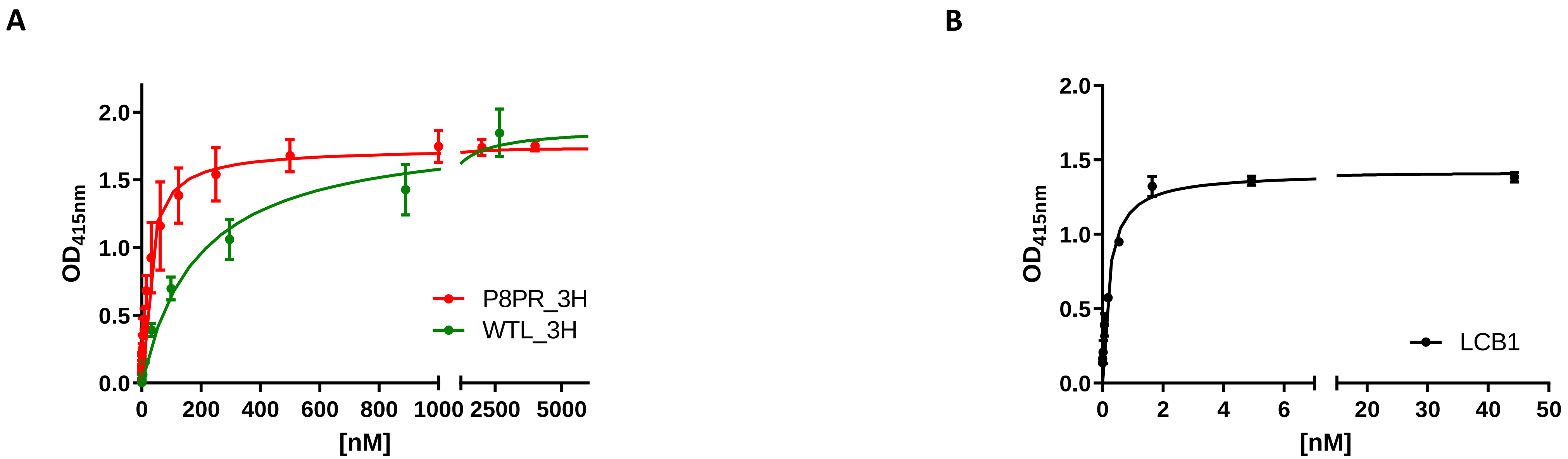

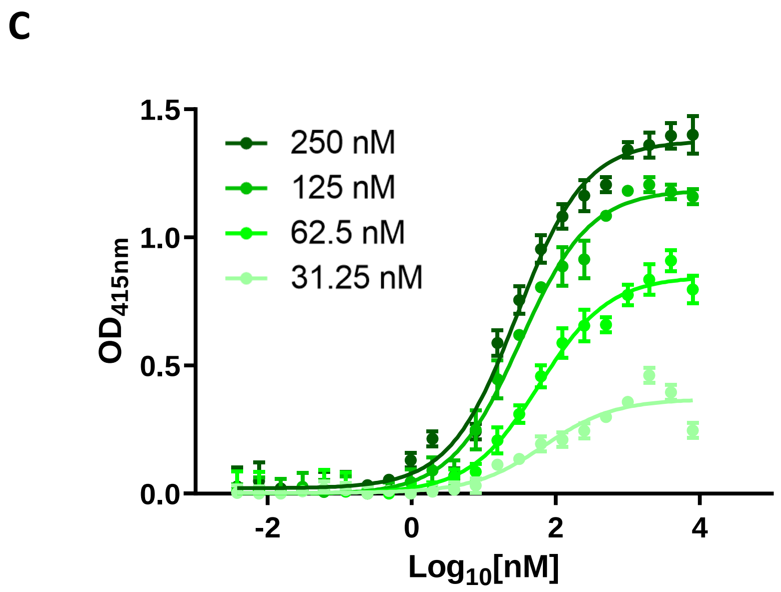

Figure S4. A. Determination of the apparent Kd values for the spike RBD of P8PR_3H (*red*) and WTL_3H (*green*). Data derived from the ELISA dose–response binding curves reported in Figure 3 were fitted to a non-linear Michaelis-Menten curve to calculate apparent Kd values (reported in Table 1). **B.** Non-linear Michaelis-Menten fitting of ELISA data for LCB1, shown in a separate graph due to the considerably higher RBD affinity of the LCB1 mini-protein (see Table 1). **C**. Determination of the apparent affinity constant of P8PR_3H for the spike RBD by ELISA dose–response assays. The concentration-dependent binding of P8PR_3H was measured in plates coated with varying RBD concentrations (250, 125, 62.5, 31.25 nM). A nonlinear log(agonist) vs. response-Variable slope curve fitting applied to the experimental data (mean ±SD of three independent replicates) yielded the following EC50 values: 29.36, 32.15, 59.53, 68.8 nM from the highest to the lowest RBD concentration.

Table S1

Nucleotide and amino acid sequences of the ACE2 mimicking polypeptides employed in this study. The sequences of the ACE2-mimicking polypeptides are in *red*. The 6XHis tag sequence is in *green*, the TEV protease recognition sequence is in *cornflower blue*, the thrombin cleavage site is in *orange*, the c-myc tag is in *purple*. The amino acid sequences of the core ACE2-mimicking polypeptides are shown in *bold red*. The monomeric P8PR_1H polypeptide utilized in this work (underlined sequences) was isolated after cleavage from the dimeric form of the polypeptide, as described in 'Materials and Methods'.

Name	Nucleotide sequence	Amino acid sequence
**P8PR_3H**	ATGGGAAGTTCACATCACCATCACCACCACAGCAGCGGTGAAAACCTGTATTTCC<br>AGTCAGGTGGCGGGTCAGCTCTAGAGGAACAATTAAAATACTTTTTAGACAAGTT<br>CATGCACGAGCTGGAAGATCTGCTGTACCAGTTGGCTTTGGCGAGCTGGAATTAT<br>AACACGAACATCACTGAGGAGAATGTTCAGAACATGAATAACGCCGGTGACAAGT<br>GGAGCGCGTTCCTGAAGGAGCAATCCACCACTGCGCAGATGTATCCGCTGCAAGA<br>GATCCAAAACCAAACGGTGAAACAACAGTTGCAGGCATTGCAAGGTGGTCTGGTA<br>CCGAGAGGCTCCGGCGGTTCCGAGCAGAAACTGATCAGCGAAGAGGATCTCTAAT<br>AACTC	**MGSSHHHHHHSSGENLYFQSGGGSALEEQLKYFLDKFMHELEDLLYQLALASWNYNTNITEENV<br>QNMNNAGDKWSAFLKEQSTTAQMYPLQEIQNQTVKQQLQALQGGLVPRGSGGSEQKLISEEDL**
**WTL_3H**	ATGAGCACCATCGAGGAACAAGCGAAGACCTTCCTGGACAAATTTAACCACGAGG<br>CGGAAGATCTGTTCTACCAGAGCAGCCTGGCGAGCTGGAACTATAACACCAACAT<br>TACCGAGGAAAACGTTCAAAACATGAACAACGCGGGTGACAAGTGGAGCGCGTTT<br>CTGAAAGAGCAGAGCACCCTGGCGCAAATGTACCCGCTGCAGGAAATCCAGAACC<br>AAACCGTTAAACAGCAACTGCAGGCGCTGCAAGGTGGCGGTGGCAGCGAACAAAA<br>ACTCATCTCAGAAGAGGATCTGAATCTCGAGCACCACCACCACCACCAC	**MSTIEEQAKTFLDKFNHEAEDLFYQSSLASWNYNTNITEENVQNMNNAGDKWSAFLKEQSTLAQ<br>MYPLQEIQNQTVKQQLQALQGGGGSEQKLISEEDLNLEHHHHHH**
**P8PR_1H**	ATGGGAAGTTCACATCACCATCACCACCACAGCAGCGGTGGCGGTGGCGGCGGCT<br>CGGGTGGTGGCGGGTCAGCTCTAGAGGAACAATTAAAATACTTTTTAGACAAGTT<br>CATGCACGAGCTGGAAGATCTGCTGTACCAGTTGGCTTTGAGCTCTGGTGGCAGT<br>GGTGGTTCCGGCTCCGGCGGCTCTGAAAACCTGTATTTCCAG**TCA**GGCGGCGGCG<br>GATCCAGCGCGCTGGAAGAACAGCTTAAGTACTTTCTGGATAAATTCATGCATGA<br>ACTGGAGGACCTGCTGTATCAGCTGGCGCTGAGCTCCCTGGTTCCGCGCGGTTCC<br>GGTGGCAGCGAGCAAAAACTGATTAGCGAGGAAGATCTG	**MGSSHHHHHHSSGGGGGGSGGGGSALEEQLKYFLDKFMHELEDLLYQLALSSGGSGGSGSGGSE<br>NLYFQSGGGGSSALEEQLKYFLDKFMHELEDLLYQLALSSLVPRGSGGSEQKLISEEDL**
**LCB1**	ATGGGAAGTTCACATCACCATCACCACCACAGCAGCGAGCAGAAACTGATCAGCG<br>AAGAGGATCTCGGTGAAAACCTGTATTTCCAGTCAGGTGACAAGGAGTGGATTCT<br>GCAAAAAATCTACGAAATTATGCGTCTGCTGGATGAGCTGGGTCACGCGGAAGCG<br>AGCATGCGTGTGAGCGACCTGATTTATGAaTTCATGAAGAAAGGCGATGAACGTC<br>TGCTGGAGGAAGCGGAGCGTCTGCTGGAGGAAGTTGAACGT	**MGSSHHHHHHSSEQKLISEEDLGENLYFQSGDKEWILQKIYEIMRLLDELGHAEASMRVSDLIY<br>EFMKKGDERLLEEAERLLEEVER**

Table S2

Half maximal inhibitory concentrations (IC_{50}) values of different engineered ACE2-derived fragments and the LCB1 mini-protein against infection by various SARS-CoV-2 spike-displaying virus like particles variants (Wuhan-Hu-1, Omicron BA.2, Omicron BQ.1.1). The IC_{50} values, expressed in µM, were calculated from neutralization assay curves reported in Figure 4 (See Materials and Methods for details).

**SARS-CoV-2 Variant**	**P8PR_3H**	**WTL_3H**	**LCB1**
Wuhan-Hu-1	0.65	N.D.*	<5 nM**
Omicron BA.2	5.30	N.D.*	0.87
Omicron BQ1.1	1.81	N.D.*	27.99

*N.D. Not detected. The neutralization inhibitory values of the samples analyzed were too low to calculate an IC_{50} value.

**The IC_{50} value was not calculated since the neutralization inhibitory values by LCB1 mini-protein were 100% at all concentration tested. Therefore the actual IC_{50} value is lower than the lowest concentration of LCB1 mini-protein tested (5nM).
